# Centile Curves and Reference Values for Height, Body Mass, Body Mass Index and Waist Circumference of Peruvian Children and Adolescents

**DOI:** 10.3390/ijerph120302905

**Published:** 2015-03-09

**Authors:** Alcibíades Bustamante, Duarte Freitas, Huiqi Pan, Peter T. Katzmarzyk, José Maia

**Affiliations:** 1 National University of Education Enrique Guzmán y Valle, Av. Guzmán y Valle s/n La Cantuta-Chosica, Lima, Peru; E-Mail: huanta2609@yahoo.es; 2 CIFI^2^D, Kinanthropometry Lab, Faculty of Sport, University of Porto, Rua Dr. Plácido Costa 91, Porto 4200-450, Portugal; 3 Department of Physical Education and Sports, University of Madeira, Colégio dos Jesuítas-Rua dos Ferreiros, Funchal 9000-082, Portugal; E-Mail: dfreitas3@uma.pt; 4 MCR Centre of Epidemiology for Child Health, UCL Institute of Child Health 30 Guilford Street London WC1N 1EH, UK; E-Mail: h.pan@ucl.ac.uk; 5 Pennington Biomedical Research Center, Louisiana State University, 6400 Perkins Rd., Baton Rouge, LA 70808, USA; E-Mail: Peter.Katzmarzyk@pbrc.edu

**Keywords:** centile curves, reference values, schoolchildren

## Abstract

This study aimed to provide height, body mass, BMI and waist circumference (WC) growth centile charts for school-children, aged 4–17 years, from central Peru, and to compare Peruvian data with North-American and Argentinean references. The sample consisted of 8753 children and adolescents (4130 boys and 4623 girls) aged 4 to 17 years, from four Peruvian cities: Barranco, La Merced, San Ramón and Junín. Height, body mass and WC were measured according to standardized techniques. Centile curves for height, body mass, BMI and WC were obtained separately for boys and girls using the LMS method. Student t-tests were used to compare mean values. Overall boys have higher median heights than girls, and the 50th percentile for body mass increases curvilinearly from 4 years of age onwards. In boys, the BMI and WC 50th percentiles increase linearly and in girls, the increase presents a curvilinear pattern. Peruvian children are shorter, lighter and have higher BMI than their counterparts in the U.S. and Argentina; in contrast, age and sex-specific WC values are lower. Height, body mass and WC of Peruvian children increased with age and variability was higher at older ages. The growth patterns for height, body mass, BMI and WC among Peruvian children were similar to those observed in North-American and Argentinean peers.

## 1. Introduction

Growth is a multifaceted process where increases in body size lead to morphological and functional changes. This process is determined by biological factors that show a high degree of sensitivity to environmental stimuli which tempers the expression of genetic potential [1,2]. As a result, sizeable variation across populations in growth patterns exists [3].

There is a general consensus that child growth is a putative health and nutrition marker of quality of life [4,5] and, therefore, monitoring growth is an important public health task. In 2006, the World Health Organization (WHO) published normative centile charts [6] based on data from the multicentre growth reference study including highly selective samples of infants and native children from Davis (United States), Accra (Ghana), Muscat (Oman), Oslo (Norway), Pelotas (Brazil), and Delhi (India). Notwithstanding the importance of international standards, data from local samples seem to be more informative [7,8,9] and centile curves for height and body mass have been published in central and south America, namely in Argentina [10], Bolivia [11], Brazil [12,13,14], Cuba [15] and Venezuela [16].

To the best of our knowledge no national charts for height, body mass, body mass index (BMI) and waist circumference (WC) are available in Peru. Peru is a country on the central western coast of South America facing the Pacific Ocean with a territorial surface of 1.3 million km^2^, 28 million inhabitants and a population density of 23.5 inhabitants per km^2^. This large territory represents a wide range of geographic, socio-economic, ethnic and cultural conditions [17]. For simplicity, Peru can be described as having three natural regions: coast, mountain and jungle. The Peruvian population includes descendants of Amerindians, European-Spanish, Afro-Americans, Chinese, and a mixture of these groups. According to the recent classification of the International Monetary Fund, Peru belongs to a class of emerging and developing economies with a Human Development Index (HDI) of 0.74 and a life expectancy at birth of 74.2 years [17,18]. 

Since there is no proper substitute for a country having its own child growth reference data and no national growth centile charts are available in Peru, the purpose of this study was twofold: (1) to provide centile charts for height, body mass, BMI and WC for school-children, aged 4–17 years, from the central Peruvian region; and (2) to compare Peruvian data with the North-American [19,20] and Argentina [10,21,22] references.

## 2. Methods

### 2.1. Sample

**Figure 1:**
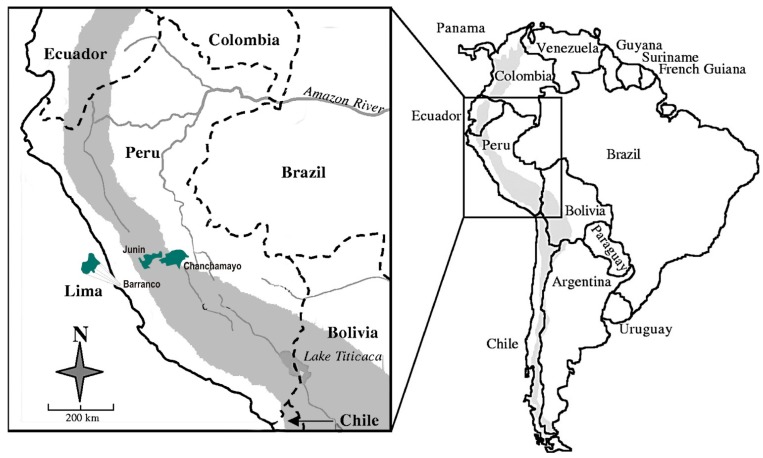
South America and representation of the region of the Andes. Location of the Peruvian cities indicated in the present study.

The participants came from the ‘Optimal and Healthy Growth Study’ (OHGS), a cross-sectional study carried out in Peru between March 2009 and June 2011. In total, 8753 children and adolescents (4130 boys and 4623 girls) aged 4 to 17 years were randomly selected from 31 public schools belonging to four cities at different altitudes: Barranco (50 m above sea level), La Merced and San Ramón (700 m and 750 m above sea level, respectively) and Junín (4130 m above sea level) (see Figure 1 and Table 1 and Table 2). All schoolchildren enrolled in the 31 schools located in the four cities were invited to participate. The response rate was 98.4%. The ethical committee of the National University of Education Enrique Guzmán y Valle and the school directors approved the OHGS. Informed consent was obtained from parents and/or legal guardians of the participants.

### 2.2. Measures

All measurements were made according to standardized techniques [23]. Height was measured to the nearest 0.1 cm with a portable stadiometer (Model ES-2060, Sanny, São Paulo, Brazil). Body mass was measured to the nearest 0.1 kg using a digital scale (Model IP68, Pesacon, Lima, Peru). BMI was obtained by the ratio of body mass to height (kg/m^2^). WC was measured with a non-stretchable fibreglass tape (model 4010, Sanny, São Paulo, Brazil) at the midpoint between the edge of the lowest rib and the superior iliac crest during shallow breathing.

**Table 1:** Geographic, socioeconomic and educational characteristics of Peru.

Characteristics			Peru
Total population			30135875
Official language and co-official			Spanish/Quechua/Aymara
Ethnic composition			Amerindians, European-Spanish
			Afro-Americans, Asiatic
Total population of school children (Regular basic education)			
		Public	5467305 (75.8%)
		Private	1746707 (24.2%)
		Urban	5709700 (79.2%)
		Rural	1504312 (20.8%)
		Boys	3701958 (51.3%)
		Girls	3512054 (48.7%)
**Geographical characteristics**			
Area (km²)			1,285,216.20
Population density (people/km²)			23.5
Altitude (m)			0–6768
**Socioeconomic characteristics**			
Human Development Index (HDI)			0.74
Life expectancy at birth (years)			74.2
Education (%) ^1^			85.7
Literacy (%) ^2^			92.9
Per capita family income (NS per month)			374.1
Primary production			Mining/Fishery/Trade/Tourism
			Agriculture/Stockbreeding

Notes: ^1^ School age population that attends school. ^2^ Person of 15 or more years who can read and write.

**Table 2:** Number of school children in the three areas of central Peru according to age and sex.

Age	Sea Level		Rainforest Area		High Altitude		Total	
	Barranco		Chanchamayo		Junín			
**(years)**	**Girls**	**Boys**	**Girls**	**Boys**	**Girls**	**Boys**	**Girls**	**Boys**
4	91	90	123	92	24	23	238	205
5	84	69	128	139	45	36	257	244
6	104	91	149	146	45	52	298	289
7	76	52	140	192	55	47	271	291
8	82	64	178	174	47	60	307	298
9	119	76	187	184	65	76	371	336
10	85	84	218	189	70	71	373	344
11	111	114	193	189	73	55	377	358
12	92	119	237	144	134	90	463	353
13	69	64	212	144	76	92	357	300
14	125	102	187	120	109	102	421	324
15	110	142	150	151	98	85	358	378
16	139	82	132	118	105	84	376	284
17	60	40	41	44	55	42	156	126
Total	1347	1189	2275	2026	1001	915	4623	4130

Notes: Age classification in each group was as follows: age 4 is between 4.00–4.99 and the same applies for all ages from 5–17 years.

### 2.3. Data Quality Control

The field team members were first trained by experienced anthropometrists for accurate anatomical landmarks, subject positioning and measurement techniques. Secondly, a random sample of 211 children and adolescents were re-measured during the first three weeks of data collection. Technical errors of measurement (TEM), and ANOVA-based intraclass correlation coefficients (R) were used to estimate the degree of precision and the proportion of the variation in measurements. TEM and R were as follows: 0.2 cm, 0.92 (height), 0.1 kg, 0.98 (body mass) and 0.9 cm, 0.92 (WC).

### 2.4. Statistical Analyses

Height, body mass, BMI and WC centiles were obtained for boys and girls separately using the LMS method [24] implemented in the LMSchartmaker Pro version 2.54 software [25]. The LMS method assumes that the outcome variable has a normal distribution after a Box-Cox power transformation is applied. Three smoothing and specific curves for each age were obtained via penalized maximum likelihood, namely: M (median), L (Box-Cox transformation) and S (coefficient of variation). The equation to derive the centiles is the following:

C100α (t) = M (t) [1 + L (t) S (t) Zα] 1/L (t)

where Zα is the normal equivalent deviate for tail area α, C100α (t) is the centile corresponding to Zα. Equivalent degrees of freedom (edf) for L(t), M(t) and S(t) measure the complexity of each fitted curve. The appropriate number of degrees of freedom was selected on the basis of the deviance, Q-tests and worm plots following the suggestions of Royston and Wright [26], van Buuren and Fredricks [27] and Pan and Cole [25,28]. The 3rd, 10th, 25th, 50th, 75th, 90th, and 97th percentiles were chosen as age- and gender-specific reference values. The proportion of the data in the channels around the seven fitted centiles was compared to the expected values of the normal distribution in each centile, showing their closeness to the expected distribution for each of the four growth characteristics, confirming a good fit (Table 3).

Student *t*-tests were used to compare the mean values of Peruvian children with North-Americans and Argentinean counterparts. Height, body mass and BMI data from American children come from the report submitted by the Centers for Disease Control and Prevention that provides United States growth charts [19]; WC percentiles values were derived from the combination of NHANES III (1988–1994), NHANES 1999–2006, Bogalusa Heart Study (1992–1994) and Fels Longitudinal Study (1976–1996) samples [20]. The WC was measured just above the uppermost lateral border of the right ilium (NHANES) and in the other studies halfway between the lowest rib and the top edge of the iliac crest.

Height and body mass data from Argentine children came from cross-sectional studies done in La Plata and Cordova cities in 1970, and a national cross-sectional sample of high school students aged 12–19 years old and done in 1985 [10]. BMI information correspond to a cross-sectional study with 4 to 16 year olds attending public and private schools in San Salvador de Jujuy city during 1995–2000 [21]. The WC values come from a cross-sectional study carried out in eight primary schools in Buenos Aires in 2003, and was measured at the umbilicus level [22]. Significance level was set at *p* < 0.05. These data were analyzed using IBM SPSS 20.

**Table 3:** Distribution of Z-score of height, body mass, BMI and waist circumference for the Peru sample compared to expectation assuming normality-area between adjacent centiles (%).

Centile	Expected (%)	Height (%)			Body mass (%)			BMI (%)			WC (%)	
		Girls	Boys		Girls	Boys		Girls	Boys		Girls	Boys
		(n = 4587)	(n = 4090)		(n = 4586)	(n = 4098)		(n = 4579)	(n = 4090)		(n = 4588)	(n = 4093)
3	3	3.0	2.8		2.9	3.2		2.8	2.9		3.0	3.4
10	7	7.7	7.4		7.7	6.7		7.0	7.3		7.5	6.4
25	15	14.7	15.2		14.7	15.9		15.7	14.9		15.1	15.4
50	25	24.8	24.5		24.2	24.1		24.7	25.6		24.6	25.8
75	25	24.8	25.0		24.9	24.6		24.6	24.2		24.7	24.5
90	15	14.4	14.5		14.5	14.7		14.1	13.5		13.6	13.3
97	7	7.5	7.3		7.8	7.3		7.8	8.1		7.9	7.5

## 3. Results

**Figure 2:**
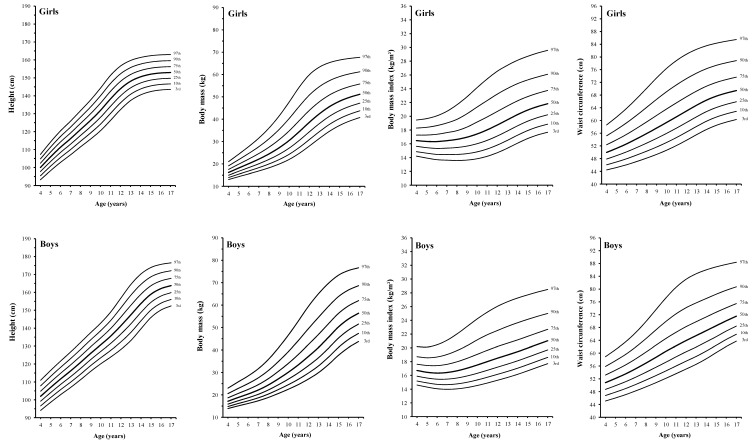
Smoothed reference curves for the 3rd, 10th, 25th, 50th, 75th, 90th and 97th percentiles for height, body mass, body mass index and waist circumference in 4 to 17 year-old Peruvian boys and girls.

Age- and gender-specific values for the 3rd, 10th, 25th, 50th, 75th, 90th, and 97th centiles are presented in Figure 2 and Table 4 and Table 5. The medians for height increase linearly from 4 to 14 years in boys and from 4 to 11 years in girls, and gradually level off, reaching a value of 165 cm in boys and 153 cm in girls. The increase is higher in boys than in girls. The P50 values for body mass increase curvilinearly from 4 years of age onward; the increase is stepper from 11 to 14 years in boys and from 9 to 11 years in girls. Maximum values are achieved at 17 years for girls (52.3 kg) and boys (58.5 kg).

Smoothed centiles for BMI and WC are presented in Figure 2 and Table 5. The BMI shows a decline from 4 to 6 years of age and then increases linearly in boys and curvilinearly in girls. At 17 years of age, the BMI of boys and girls are 22.2 kg/m^2^ and 21.5 kg/m^2^, respectively. The variability increases with increasing age. As for the BMI, the P50 values for WC increase linearly in boys and curvilinearly in girls through 4 to 17 years of age. The median for WC at 17 years old is 72.8 cm in boys and 70.1 cm in girls. Variability of WC also increases with age and is higher at 17 years old.

**Figure 3:**
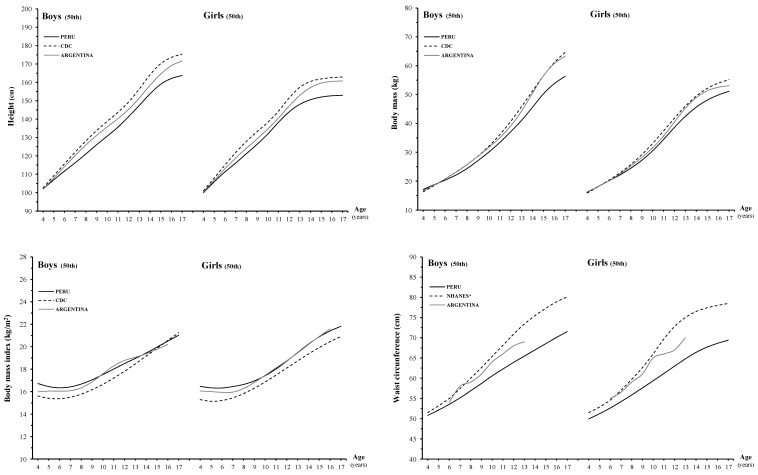
Comparison of the age and sex specific 50th percentile values for height, body mass, body mass index and waist circumference between Peruvian boys and girls and those from North-America and Argentina.

Figure 3 displays the 50th centiles for height, body mass, BMI and WC of Peruvian children against North-American [19,20], and Argentinean [10,21,22] counterparts. Table 6 and Table 7 provides means, mean differences and *p* values for these growth characteristics. The Peruvian boys are shorter than North-American and Argentinean peers. The average difference between Peruvian and North-American boys is ~7.8 cm and between Peruvian and Argentinean is ~4.5 cm. Corresponding values for girls are ~7.0 cm and ~4.1 cm. For body mass, Peruvian boys and girls are lighter than North-American and Argentinean counterparts. The differences between Peruvian and North-American children are ~4.1 kg in boys and ~2.8 kg in girls. The differential between Peruvian and Argentinean children is ~3.4 kg (boys) and 1.6 kg (girls). For BMI, Peruvian boys have higher mean values than North-American peers and the difference is ~0.8 kg/m^2^. In girls, the difference is ~0.9 kg/m^2^ in the age interval 4–17 years. The differential between Peruvian and Argentinean boys and girls is ~0.4 kg/m^2^ between 4 and 9 years of age. The WC of Peruvian children is lower than North-Americans and Argentinean peers in all age intervals. Differences between Peruvian and North-American children are ~5.4 cm in boys and ~6.6 cm in girls. The same differential between Peruvian and Argentinean children is ~2.8 cm in boys and ~3.9 cm in girls between 6 and 13 years of age.

**Table 4:** Age- and sex-specific percentiles of height (cm) and body mass (kg) for school-aged Peruvian children and adolescents.

Age			Centiles: Height (cm)									Centiles: Body Mass (kg)						
(Years)	L	S	3rd	10th	25th	50th (M)	75th	90th	97th	L	S	3rd	10th	25th	50th (M)	75th	90th	97th
**Boys**
4	−0.5772	0.0412	94.1	96.6	99.2	102.0	104.8	107.8	111.0	−1.3460	0.1214	13.9	14.8	15.9	17.1	18.7	20.6	23.0
5	−0.5772	0.0415	98.6	101.3	104.1	107.0	110.0	113.1	116.5	−1.3184	0.1286	15.1	16.1	17.3	18.8	20.6	22.8	25.7
6	−0.5772	0.0419	102.9	105.7	108.6	111.7	114.9	118.2	121.7	−1.2921	0.1356	16.1	17.3	18.7	20.3	22.4	25.0	28.4
7	−0.5772	0.0425	107.0	109.9	113.0	116.2	119.6	123.1	126.8	−1.2623	0.1433	17.3	18.6	20.2	22.1	24.5	27.5	31.6
8	−0.5772	0.0428	111.3	114.4	117.6	121.0	124.6	128.2	132.1	−1.2256	0.1525	18.8	20.3	22.1	24.3	27.1	30.8	35.7
9	−0.5772	0.0426	116.0	119.2	122.5	126.0	129.7	133.5	137.5	−1.1817	0.1625	20.6	22.3	24.5	27.1	30.4	34.8	40.8
10	−0.5772	0.0433	120.2	123.5	127.1	130.8	134.6	138.7	142.9	−1.1326	0.1724	22.5	24.5	27.0	30.1	34.0	39.2	46.5
11	−0.5772	0.0461	124.0	127.7	131.6	135.6	139.9	144.4	149.1	−1.0777	0.1813	24.6	26.9	29.8	33.4	38.0	44.1	52.8
12	−0.5772	0.0504	128.2	132.3	136.7	141.3	146.2	151.4	156.8	−1.0165	0.1870	27.0	29.7	33.0	37.1	42.4	49.4	59.4
13	−0.5772	0.0527	133.1	137.7	142.4	147.5	152.8	158.5	164.4	−0.9506	0.1861	29.8	32.9	36.5	41.1	46.8	54.5	65.0
14	−0.5772	0.0496	139.6	144.1	148.8	153.8	159.0	164.5	170.3	−0.8806	0.1771	33.5	36.8	40.7	45.6	51.6	59.4	69.7
15	−0.5772	0.0438	146.0	150.1	154.4	159.0	163.7	168.7	173.9	−0.8112	0.1628	37.6	41.1	45.3	50.2	56.3	63.8	73.3
16	−0.5772	0.0392	150.1	154.0	158.0	162.1	166.4	170.9	175.6	−0.7498	0.1493	41.1	44.7	48.9	53.8	59.6	66.7	75.4
17	−0.5772	0.0366	152.4	156.1	159.8	163.8	167.9	172.1	176.5	−0.6968	0.1383	43.8	47.4	51.5	56.4	62.0	68.7	76.6
**Girls**																					
4	0.0064	0.0352	93.2	95.4	97.7	100.0	102.4	104.8	107.3	−0.8235	0.1180	13.0	13.9	15.0	16.2	17.5	19.1	21.0
5	0.0064	0.0377	98.2	100.7	103.2	105.9	108.6	111.3	114.2	−0.8235	0.1303	14.3	15.4	16.7	18.1	19.9	21.9	24.3
6	0.0064	0.0395	102.8	105.6	108.4	111.3	114.2	117.3	120.4	−0.8235	0.1422	15.6	16.8	18.3	20.1	22.2	24.7	27.8
7	0.0064	0.0405	107.0	109.9	112.9	116.0	119.2	122.5	125.8	−0.8235	0.1539	16.8	18.3	20.1	22.1	24.6	27.7	31.6
8	0.0064	0.0416	111.5	114.6	117.8	121.1	124.5	128.0	131.6	−0.8235	0.1659	18.2	20.0	22.0	24.5	27.5	31.2	36.0
9	0.0064	0.0430	115.8	119.2	122.7	126.2	129.9	133.7	137.6	−0.8235	0.1777	19.9	21.9	24.3	27.2	30.8	35.4	41.4
10	0.0064	0.0450	120.5	124.2	128.0	131.8	135.9	140.0	144.3	−0.8235	0.1872	22.0	24.3	27.1	30.5	34.8	40.3	47.7
11	0.0064	0.0453	126.2	130.1	134.1	138.2	142.5	146.8	151.4	−0.8235	0.1904	24.7	27.3	30.5	34.4	39.4	45.8	54.4
12	0.0064	0.0422	132.3	136.0	139.9	143.9	148.0	152.2	156.6	−0.8235	0.1843	28.0	30.9	34.3	38.6	44.0	50.8	59.9
13	0.0064	0.0378	137.3	140.8	144.4	148.1	151.8	155.7	159.7	−0.8235	0.1706	31.4	34.5	38.1	42.4	47.8	54.6	63.3
14	0.0064	0.0347	140.5	143.8	147.1	150.6	154.1	157.7	161.4	−0.8235	0.1553	34.6	37.7	41.3	45.6	50.9	57.3	65.3
15	0.0064	0.0329	142.3	145.5	148.7	152.0	155.4	158.8	162.3	−0.8235	0.1424	37.2	40.3	43.9	48.1	53.1	59.1	66.5
16	0.0064	0.0321	143.2	146.3	149.5	152.7	156.0	159.4	162.8	−0.8235	0.1326	39.2	42.2	45.7	49.8	54.6	60.3	67.2
17	0.0064	0.0318	143.6	146.6	149.8	153.0	156.2	159.6	163.0	−0.8235	0.1251	40.7	43.8	47.2	51.2	55.8	61.2	67.7

Notes: Age: completed age, e.g., 4 years = 4.00–4.99 years.

**Table 5:** Age- and sex-specific percentiles of body mass index (kg/m²) and waist circumference (cm) for school-aged Peruvian children and adolescents.

Age			Centiles: Body Mass Index (kg/m²)									Centiles: Waist Circumference (cm)						
(Years)	L	S	3rd	10th	25th	50th (M)	75th	90th	97th	L	S	3rd	10th	25th	50th (M)	75th	90th	97th
**Boys**	
4	−2.2535	0.0753	14.6	15.2	15.8	16.7	17.6	18.7	20.2	−1.5809	0.0660	45.1	46.8	48.7	50.8	53.2	55.9	58.9
5	−2.2535	0.0824	14.3	14.9	15.6	16.4	17.4	18.6	20.1	−1.7335	0.0694	46.0	47.8	49.9	52.1	54.7	57.7	61.1
6	−2.2535	0.0896	14.1	14.7	15.4	16.3	17.4	18.7	20.5	−1.8922	0.0730	47.1	49.0	51.1	53.5	56.3	59.6	63.5
7	−2.2535	0.0972	14.0	14.7	15.5	16.4	17.6	19.0	21.1	−2.0652	0.0772	48.2	50.2	52.5	55.1	58.2	61.9	66.4
8	−2.2535	0.1048	14.0	14.8	15.6	16.7	18.0	19.6	22.0	−2.2483	0.0820	49.5	51.6	54.0	56.9	60.3	64.5	69.8
9	−2.2535	0.1113	14.2	15.0	15.9	17.1	18.5	20.3	23.1	−2.4370	0.0867	50.8	53.0	55.6	58.7	62.5	67.2	73.5
10	−2.2535	0.1160	14.5	15.3	16.3	17.5	19.1	21.2	24.2	−2.6247	0.0903	52.2	54.5	57.2	60.6	64.6	69.9	77.2
11	−2.2535	0.1186	14.9	15.7	16.7	18.0	19.6	21.9	25.2	−2.8058	0.0920	53.6	56.0	58.8	62.3	66.5	72.2	80.5
12	−2.2535	0.1191	15.3	16.2	17.2	18.5	20.2	22.5	26.0	−2.9769	0.0917	55.0	57.5	60.4	63.9	68.3	74.2	83.0
13	−2.2535	0.1177	15.7	16.6	17.6	19.0	20.7	23.1	26.7	−3.1406	0.0895	56.5	59.0	61.9	65.5	69.8	75.7	84.8
14	−2.2535	0.1150	16.2	17.0	18.1	19.4	21.1	23.6	27.2	−3.3097	0.0859	58.2	60.6	63.5	67.0	71.3	77.0	86.0
15	−2.2535	0.1117	16.7	17.6	18.6	20.0	21.7	24.1	27.7	−3.4879	0.0811	60.2	62.4	65.1	68.5	72.8	78.4	86.9
16	−2.2535	0.1083	17.2	18.1	19.2	20.5	22.2	24.6	28.1	−3.6608	0.0765	62.1	64.3	66.9	70.1	74.2	79.6	87.7
17	−2.2535	0.1049	17.7	18.6	19.7	21.0	22.7	25.0	28.5	−3.8214	0.0725	63.7	65.9	68.4	71.5	75.5	80.7	88.4
**Girls**																					
4	−1.2523	0.0784	14.2	14.9	15.6	16.5	17.3	18.3	19.5	−2.2787	0.0670	44.4	46.0	47.9	49.9	52.3	55.2	58.6
5	−1.2523	0.0889	13.9	14.6	15.4	16.3	17.3	18.4	19.7	−2.2787	0.0725	45.2	46.9	48.9	51.2	53.9	57.1	61.1
6	−1.2523	0.0982	13.7	14.5	15.3	16.3	17.4	18.6	20.0	−2.2787	0.0780	46.1	47.9	50.1	52.6	55.6	59.3	63.8
7	−1.2523	0.1066	13.6	14.4	15.4	16.4	17.6	19.0	20.7	−2.2787	0.0834	47.1	49.1	51.4	54.2	57.5	61.6	66.9
8	−1.2523	0.1156	13.6	14.5	15.5	16.6	17.9	19.5	21.7	−2.2787	0.0886	48.1	50.3	52.8	55.9	59.5	64.1	70.1
9	−1.2523	0.1255	13.6	14.6	15.7	17.0	18.4	20.4	22.8	−2.2787	0.0931	49.3	51.6	54.3	57.6	61.6	66.6	73.4
10	−1.2523	0.1341	13.8	14.8	16.0	17.4	19.2	21.3	24.2	−2.2787	0.0959	50.6	53.1	55.9	59.4	63.6	69.0	76.4
11	−1.2523	0.1397	14.2	15.2	16.5	18.0	19.9	22.3	25.4	−2.2787	0.0965	52.1	54.6	57.6	61.2	65.6	71.2	78.9
12	−1.2523	0.1413	14.7	15.8	17.1	18.7	20.7	23.2	26.5	−2.2787	0.0953	53.8	56.3	59.4	63.0	67.5	73.2	80.9
13	−1.2523	0.1386	15.4	16.5	17.8	19.5	21.5	24.0	27.4	−2.2787	0.0926	55.5	58.1	61.1	64.8	69.2	74.9	82.4
14	−1.2523	0.1332	16.1	17.2	18.6	20.2	22.2	24.7	28.1	−2.2787	0.0894	57.1	59.7	62.8	66.4	70.8	76.3	83.5
15	−1.2523	0.1271	16.7	17.9	19.3	20.9	22.8	25.3	28.7	−2.2787	0.0866	58.5	61.1	64.1	67.7	72.0	77.4	84.3
16	−1.2523	0.1227	17.3	18.4	19.8	21.4	23.3	25.7	29.2	−2.2787	0.0845	59.5	62.1	65.1	68.6	72.9	78.2	85.0
17	−1.2523	0.1205	17.7	18.8	20.2	21.8	23.7	26.1	29.6	−2.2787	0.0829	60.3	62.9	65.9	69.4	73.6	78.8	85.5

Note: Age: completed age, e.g., 4 years = 4.00–4.99 years.

**Table 6:** Results of mean differences between the CDC and Peruvian schoolchildren for height, body mass, body mass index and waist circumference from both sexes aged 4 to 17 years.

Age(Years)		Height					Body Mass					Body Mass Index					Waist Circumference			
		CDC	PERU	Mean	*p*		CDC	PERU	Mean	*p*		CDC	PERU	Mean	*p*		NHNES	PERU	Mean	*p*
		Mean	Mean	Difference		**	Mean	Mean	Difference		**	Mean	Mean	Difference			Mean	Mean	Difference	
**Boys**																				
4		105.6	104.7	−0.86	0.010		17.7	18.2	0.56	0.003		15.8	16.6	0.78	0.001		51.5	52.3	0.75	0.008
5		112.4	109.6	−2.85	0.001		20.0	20.2	0.20	0.347		15.8	16.7	0.99	0.001		53.2	53.3	0.63	0.837
6		118.7	113.5	−5.15	0.001		22.2	21.7	−0.52	0.022		15.8	16.8	1.02	0.001		55.0	54.8	−0.16	0.532
7		125.0	119.1	−5.97	0.001		24.8	24.2	−0.61	0.026		15.9	17.0	1.06	0.001		57.4	56.9	−0.52	0.093
8		130.1	123.5	−6.63	0.001		27.4	26.7	−0.74	0.027		16.2	17.4	1.14	0.001		60.0	58.9	−1.10	0.003
9		135.8	128.8	−6.99	0.001		31.2	29.9	−1.27	0.001		16.9	17.9	1.00	0.001		62.6	60.9	−1.66	0.001
10		141.0	133.0	−7.95	0.001		34.8	32.7	−2.06	0.001		17.5	18.3	0.86	0.001		65.4	62.4	−3.73	0.001
11		146.1	138.5	−7.64	0.001		39.0	37.4	−1.56	0.001		18.2	19.4	1.17	0.001		68.1	65.2	−2.91	0.001
12		153.1	145.1	−8.02	0.001		43.5	42.0	−1.51	0.010		18.6	19.7	1.09	0.001		70.9	66.8	−4.14	0.001
13		159.7	150.1	−9.65	0.001		49.8	44.5	−5.35	0.001		19.5	19.6	0.07	0.708		73.4	66.5	−7.71	0.001
14		167.3	156.8	−10.44	0.001		56.4	50.1	−6.33	0.001		20.2	20.2	0.25	0.878		75.5	68.8	−6.73	0.001
15		171.5	160.8	−10.67	0.001		61.1	53.8	−7.29	0.001		20.8	20.7	−0.03	0.830		77.3	70.2	−7.11	0.001
16		174.5	163.1	−11.33	0.001		66.2	56.5	−9.78	0.001		21.7	21.1	−0.58	0.001		78.9	72.0	−6.87	0.001
17		175.9	163.2	−12.73	0.001		67.8	57.4	−10.32	0.001		21.9	21.5	−0.38	0.096		80.1	72.6	−7.50	0.001
**Girls**																				
4		104.5	103.5	−1.01	0.002		17.2	17.5	0.30	0.067		15.7	16.3	0.63	0.001		51.5	51.1	−0.44	0.103
5		111.8	109.3	−2.50	0.001		19.8	19.9	0.12	0.580		15.7	16.6	0.84	0.001		52.9	52.2	−0.65	0.028
6		117.9	113.0	−4.86	0.001		21.6	21.3	−0.29	0.190		15.5	16.6	1.14	0.001		54.6	53.9	−0.67	0.013
7		123.9	118.5	−5.38	0.001		24.3	24.0	−0.28	0.313		15.8	17.0	1.20	0.001		57.0	55.8	−1.20	0.001
8		129.9	124.3	−5.55	0.001		27.6	26.7	−0.95	0.002		16.4	17.2	0.79	0.001		59.7	57.4	−2.26	0.001
9		135.6	129.8	−5.79	0.001		31.7	30.4	−1.26	0.001		17.2	17.9	0.66	0.001		62.6	60.0	−2.64	0.001
10		141.4	135.2	−6.19	0.001		34.9	33.2	−1.71	0.001		17.4	18.0	0.54	0.001		66.0	60.7	−5.34	0.001
11		148.3	142.0	−6.24	0.001		40.8	39.1	−1.75	0.001		18.6	19.2	0.61	0.001		69.7	63.4	−6.31	0.001
12		154.7	145.9	−8.88	0.001		46.4	41.8	−4.58	0.010		19.3	19.5	0.21	0.140		72.8	64.4	−8.37	0.001
13		159.0	149.6	−9.41	0.001		51.1	45.7	−5.32	0.001		20.2	20.4	0.21	0.198		75.0	66.8	−8.25	0.001
14		161.0	151.0	−14.00	0.001		54.4	48.2	−6.21	0.001		21.0	21.1	0.13	0.397		76.5	68.1	−8.39	0.001
15		163.0	152.3	−10.66	0.001		56.1	50.1	−6.02	0.001		21.0	21.5	0.50	0.001		77.4	69.0	−8.39	0.001
16		162.6	153.0	−9.54	0.001		57.5	51.6	−5.94	0.001		21.8	22.0	0.23	0.120		78.0	69.8	−8.21	0.001
17		163.0	152.9	−10.03	0.001		59.0	52.5	−6.53	0.001		22.3	22.4	0.16	0.543		78.5	70.5	−7.95	0.001

**Table 7:** Results of mean differences between Argentina and Peruvian schoolchildren for height, body mass, body mass index and waist circumference from both sexes aged 4 to 17 years.

Age(Years)		Height					Body Mass					Body Mass Index					Waist Circumference			
		Argentina	Peru	Mean	*p*		Argentina	Peru	Mean	*p*		Argentina	Peru	Mean	*p*		Argentina	Peru	Mean	*p*
		Mean	Mean	Difference			Mean	Mean	Difference		**	Mean	Mean	Difference			Mean	Mean	Difference	
**Boys**																				
4		101.9	104.7	2.84	0.001		16.7	18.2	1.49	0.001		16.0	16.6	0.59	0.001					
5		107.9	109.6	1.64	0.001		18.7	20.2	1.52	0.001		16.0	16.7	0.70	0.001					
6		114.2	113.5	−0.60	0.056		20.7	21.7	0.98	0.001		16.0	16.8	0.73	0.001		54.0	54.8	0.84	0.001
7		120.2	119.1	−1.17	0.001		23.1	24.2	1.14	0.001		16.1	17.0	0.88	0.001		58.0	56.9	−1.12	0.001
8		125.9	123.5	−2.44	0.001		25.7	26.7	1.02	0.002		16.3	17.4	1.05	0.001		59.0	58.9	−0.10	0.785
9		131.1	128.8	−2.25	0.001		28.6	29.9	1.35	0.001		16.9	17.9	1.07	0.001		61.0	60.9	−0.06	0.876
10		135.8	133.0	−2.75	0.001		31.7	32.7	1.03	0.011		17.6	18.3	0.76	0.001		64.0	62.4	−1.57	0.001
11		140.3	138.5	−1.80	0.001		35.0	37.4	2.40	0.001		18.3	19.4	1.06	0.001		66.0	65.2	−0.81	0.056
12		145.4	145.1	−0.26	0.572		39.1	42.0	2.88	0.001		18.8	19.7	0.90	0.001		68.0	66.8	−1.24	0.006
13		151.5	150.1	−1.44	0.006		44.3	44.5	0.19	0.745		19.1	19.6	0.50	0.005		69.0	66.5	−2.48	0.001
14		158.4	156.8	−1.56	0.001		50.5	50.1	−0.36	0.540		19.4	20.2	0.84	0.001					
15		164.6	160.8	−3.74	0.001		56.5	53.8	−2.71	0.001		19.8	20.7	0.95	0.001					
16		169.1	163.1	−5.96	0.001		60.8	56.5	−4.31	0.001		20.2	21.1	0.95	0.001					
17		171.7	163.2	−8.48	0.001		63.3	57.4	−5.86	0.001										
**Girls**																				
4		100.5	103.5	3.00	0.001		16.3	17.5	1.19	0.001		16.1	16.3	0.24	0.022					
5		106.7	109.3	2.62	0.001		18.1	19.9	1.79	0.001		16.0	16.6	0.55	0.001					
6		113.0	113.0	0.04	0.896		20.1	21.3	1.27	0.001		15.9	16.6	0.69	0.001		55.0	53.9	−1.07	0.001
7		118.8	118.5	−0.27	0.426		22.5	24.0	1.56	0.001		16.0	17.0	1.04	0.001		56.5	55.8	−0.70	0.025
8		124.1	124.3	0.20	0.571		25.2	26.7	1.46	0.001		16.3	17.2	0.89	0.001		59.0	57.4	−1.56	0.001
9		129.2	129.8	0.55	0.116		28.2	30.4	2.25	0.001		16.8	17.9	1.06	0.001		61.0	60.0	−1.04	0.005
10		134.6	135.2	0.62	0.109		31.5	33.2	1.64	0.001		17.5	18.0	0.51	0.001		65.0	60.7	−4.34	0.001
11		140.6	142.0	1.47	0.001		35.6	39.1	3.45	0.001		18.1	19.2	1.06	0.001		66.0	63.4	−2.61	0.001
12		147.0	145.9	−1.18	0.001		40.5	41.8	1.28	0.002		18.8	19.5	0.77	0.001		67.0	64.4	−2.57	0.001
13		152.9	149.6	−3.29	0.001		45.2	45.7	0.49	0.251		19.4	20.4	0.93	0.001		70.0	66.8	−3.25	0.001
14		157.2	151.0	−6.17	0.001		48.9	48.2	−0.71	0.089		20.2	21.1	0.90	0.001					
15		159.6	152.3	−7.26	0.001		51.3	50.1	−1.23	0.002		20.9	21.5	0.63	0.001					
16		160.5	153.0	−7.53	0.001		52.5	51.6	−0.91	0.091		21.6	22.0	0.43	0.004					
17		160.7	152.9	−7.80	0.001		53.1	52.5	−0.59	0.394										

## 4. Discussion

Since the last century, Peru, as well as other Latin American countries, are experiencing epidemiological and nutritional transitions with not only dramatic decreases in malnutrition and stunting, but also increases in obesity [29]. Furthermore, over the last decade the Peruvian population has undergone significant changes in living conditions that are highly related to the overall improvement in their economy and general health status [18]. In this context, the use of simple, reliable and valid anthropometric indicators such as height, body mass, BMI, and WC, as well as their respective centile charts, are highly valuable tools in public health surveillance and epidemiology [9].

The trends in height, body mass, BMI and WC of Peruvian children and adolescents were similar to other international data. In height and body mass, the individual variation expressed in terms of the range between the 3rd and 97th percentiles increased progressively with age through childhood and adolescence. Furthermore, there is high inter-individual variation starting at an early age and clearly expressed in the upper centiles. Additionally, the adiposity rebound starting at age 6–7 years was noted in Peruvian children and parallels the study of Rolland-Cachera *et al*. [30].

Peruvian children were shorter and lighter than North-American and Argentinean peers. One possible explanation may be rooted in genetic potential which has been previously suggested by several authors [3,10,15]. Another additional factor relies on the fact that children who were born and live in high altitudes and/or subjected to chronic environmental stress, such as those living in the forest areas, had lower height and body mass values when compared to sea level standards [31,32]. Furthermore, nutritional stress, particularly in rural populations, plays an important role in co-regulating the growth process [33], although some growth delay may be expected even when socio-economic and nutritional factors have been optimized [34,35]. It is widely accepted that BMI is a suitable anthropometric marker when assessing obesity in clinical and epidemiological settings [36], while the WC is used as a relatively consistent indicator of cardio metabolic risk [37]. The Peruvian 50th BMI centile was slightly higher than the North-American [19] and similar to Argentinean [21] reference. On the contrary, the 50th WC centile of Peruvian children was below the 50th WC centiles of North-Americans and Argentineans [20,22]. The interpretation of these results requires taking into account the morphological characteristics of the population. For example, Peruvian children have a lower relative leg length in comparison to their American peers, which directly influences their stature. This condition partly explains their higher BMI values. On the other hand, in the updating of the 2000 CDC centile charts body mass that corresponded to NHANES III was excluded in order to avoid significant modifications of BMI-for-age and body mass-for-age [19]. Children and adolescents below the 5th centile may involve those with growth delay, genetically low stature, a phenotype or thrifty genotype or a complex mixture of all these factors [38]. In the opposite, those above the 75th centile may be explained by the presence of a high prevalence of overweight and obesity in the studied population [39]. For WC, comparisons are difficult because there are no international references accepted by the scientific community. The use of different WC measurement protocols limits comparisons between countries which may affect, for example, the estimations of obesity and cardio metabolic risk factors prevalence [40]. The differences found when making comparisons with Argentine and American children can be partly explained by the use of different anatomical locations to measure WC. However, the WC increase in the preschool years may be a sign that this age is crucial for the emergence of central obesity [22].

The lower stature and body mass of Peruvian children and adolescents in relation to North-American and Argentinean counterparts may be a result of genetic, geographical, cultural and socio-economic factors [33,34,35]. However, socio-economic factors deserve further attention. It is well known that the economic situation in Peru has gradually improved in the last decade, which possibly gave rise to better life conditions to important sectors of the population [17]. However, a sustained economic growth may not be enough to quench the poverty. In 2011, 17.8% of children under the age of 5 years suffered from chronic malnutrition, 7.8% suffered from global malnutrition and 1.5% suffered from acute malnutrition; in addition, 27.6% of pregnant women suffered from anemia [41]. Notwithstanding, these rates have decreased in recent years. A 2012 report of the INS revealed a prevalence of overweight and obesity of 6.4% and 1.8%, respectively, in children less than 5 years of age [42]. It was also reported that 34.4% of pregnant women were overweight, while 12.7% had shortfalls of weight, affecting in 2011 to nearly one of every eight women [41]. Furthermore, the prevalence of overweight and obesity in Peruvian children, aged 6–11 years, was 21.7% and 7%, respectively [43].

Height, body mass, BMI and WC centile charts provided in this article are based on data from a cross-sectional study of children and adolescents who attended public schools in Peru central region. In very general terms, they provide novel and useful information for monitoring the growth of children, as well as for the identification of children that maybe at risk of obesity, stunting and wasting, and maybe related to high central obesity given WC upper centiles. It represents an advance in the perspective of having national references. Notwithstanding the relevance of the present information, at least four limitations have to be stated: (1) despite the size of our sample, it is not representative of the total children and adolescents´ Peruvian population; (2) the cross-sectional outline of this study does not allow to dynamically analyze intra-individual changes that occur throughout the growing period as a result of complex biological and environmental interactions; (3) no information is presently available about the racial/ethnic composition of Peruvian schoolchildren, and so we were not able to stratify our sample according to this condition. Yet, we are confident that the present charts reflect Peruvian children and adolescent physical growth and will be useful to clinicians and educators throughout the country; (4) although the Argentinian data are 30–45 years old, they were recently (5 years ago) updated using LMS to provide growth charts. In addition, WC is frequently measured at different anatomical landmarks which cause problems with comparisons. We ask the readers to bear this information in mind.

## 5. Conclusions

In summary, the P50 of height, body mass and WC of Peruvian children increased with age and variability was higher in the older age intervals. BMI showed a decline from 4 years to 6 years of age and then increased through 17 years. Growth patterns of Peruvian children in height, body mass, BMI and WC were similar to those observed in North-American and Argentinean peers, but differences in values were observed. Peruvian children were shorter, lighter and had higher BMI values than North-American and Argentinean counterparts. In contrast, the WC of Peruvian children was lower than North-American and Argentinean age- and sex-specific peers.
